# Leaching of Carbon Reinforced Concrete—Part 2: Discussion of Evaluation Concepts and Modelling

**DOI:** 10.3390/ma13214937

**Published:** 2020-11-03

**Authors:** Lia Weiler, Anya Vollpracht, Thomas Matschei

**Affiliations:** Institute of Building Materials Research, RWTH Aachen University, 52062 Aachen, Germany; vollpracht@ibac.rwth-aachen.de (A.V.); matschei@ibac.rwth-aachen.de (T.M.)

**Keywords:** leaching, irrigated construction elements, environmental compatibility, irrigated building materials, environmental assessment, evaluation concepts

## Abstract

Possible threats on the environment and human health by the leaching of new building materials and composites in contact to water should be prevented from the outset. It is therefore necessary to assess and ensure their environmental compatibility. For irrigated construction elements this is a challenging task, as there is no general correlation between known testing methods and outdoor emissions. A feasible assessment concept is needed for these conditions. In this work the German assessment method for permanently wet building materials is applied on different carbon reinforced concrete (C^3^) leaching data. Furthermore, emission prediction approaches of the Dutch building Materials Decree and the software COMLEAM are tested. The established methods are not yet suitable to determine the complex long term outdoor emissions of irrigated C^3^. In order to achieve realistic results in time saving testing methods and to define reasonable release limits, it is necessary to determine and verify the relevant influencing parameters on leaching through intermittent water contact. This research works out leaching patterns and correlations between inorganic substances. It is shown that the input parameters time of exposure, contact time, air temperature, air humidity, runoff and background concentration should be considered to predict the leaching processes from irrigated concrete phenomenologically.

## 1. Introduction

Following the construction products regulation (CPR), directive No 305/2011 of the European Parliament, building products must not harm the user or the environment throughout their whole life cycle [1]. The directive has to be executed directly, but member states can enact additional regulations. This theoretically leads to high requirements concerning the environmental compatibility of building products.

Substances that are harmful to the environment can be emitted during processing, service life or recycling and disposal of building products. Emissions occur in the form of particles or by outgassing into the air and by leaching into the soil and/or ground- and surface water bodies.

To protect these compartments and also human health, but also to secure future recyclability and therefore resource efficiency, contaminants in building products should remain in low ranges. However, a maximum allowable content is regulated only for a few potentially harmful substances. Furthermore, the total content is often not applicable to assess possible emissions. It is well known that material composition and leaching conditions rather than the total substances content determine the release of substances from different materials to the environment, e.g., [2,3,4,5,6,7].

Even established building materials are not to be neglected for further research on their environmental behavior. Due to the continual developing states of knowledge, precision in analytics, and legal regulations, as for example the EU POP- [8] and REACH regulations [9], commonly used products can turn out to be hazardous to human health and the environment [2,10]. Well-known cases are the carcinogenic asbestos and, more recent, the flame retardant Hexabromocyclododecane (HBCD), bearing PBT characteristics (persistent, bio accumulative, toxic) [11].

To tackle this issue, the European Commission is, based on the CPR, required to release harmonized testing standards and assessment methods. This is partially realized for some cases by EN 16516 for Volatile Organic Compounds [12], CEN/TS 16637, Part 1 to 3, for the leaching of hazardous substances from building products [13], or EN 16105:2011 for the leaching of paints and varnishes [14]. The assessment methods of the harmonized testing standards are desired to be harmonized, too, but are to date implemented nationally so that different European countries, as for example the Netherlands or Germany, developed different regulations for the release of defined substances from building products.

In Germany, an assessment concept for the leaching behavior of construction products and materials from the “Centre of Competence for Construction” (DIBt) is used in the context of technical approvals for new building materials [15]. The concept earmarks leaching tests for different materials in different application scenarios. Monolithic building materials in permanent contact with water are tested with the so-called Dynamic Surface Leaching Test (DSLT) regulated by the harmonized European technical specification CEN/TS 16637-2 [13]. The released amount of relevant substances is then compared to specific limits [16], derived from threshold values for groundwater of the German Working Group on water issues of the Federal States and the Federal Government represented by the Federal Environment Ministry (LAWA) [17].

The concept applies for construction elements in direct contact to soil. Irrigated construction elements, as for example roofs or façades, are not considered in the DIBt concept so far [18,19]; though the relevance of runoff emissions is, inter alia, shown by Wicke et al. [20], Gasperi et al. [21], Clara et al. [22] and Scherer [6] and the issue has been discussed by an expert group of the DIBt and different studies [18,23].

Emissions from intermittently wetted construction elements are difficult to predict and therefore also to assess as these materials experience a permanent wet–dry stress, which causes a deviating leaching behavior. Dry phases may lead to faster capillary transport and therefore an increased availability for leaching or wash-off in the following rain [24]. Also increased or lower release depending on the respective substance, chosen point in time, and reference value as, e.g., contact time or amount of water applied [18,25], and changing release patterns compared to permanent wet components [6,25] can be observed.

However, the Netherlands assess irrigated construction elements by using the DSLT and a transfer function that considers the reduced water contact time with a factor of 0.1 [26,27,28]. This method might not cover the worst case leaching conditions and therefore underestimates the actual emissions. Hecht and Schoknecht et al. for example showed that not only the duration of water contact, but also the transport during drying phases determines the emissions [24,29], the DSLT does not achieve realistic exposure conditions [30]. Still it is desirable to find or develop a horizontal and therefore universally applicable and easily adaptable concept for the assessment of the environmental compatibility of irrigated construction components. This concept could be used to recognize possible threats from the outset, and ensure a sustainable application of new building materials and composites.

In order to achieve realistic results in time saving testing methods and to define reasonable release limits, it is necessary to determine and verify the relevant influencing parameters on leaching through intermittent water contact and other relevant environmental factors on the respective material and therefore also to create a wider database [5,25,29].

In part 1 of this study [25] the leaching behavior of carbon textile reinforced concrete (C^3^) was investigated under two established laboratory tests: DSLT and pH-dependent leaching, an artificial indoor irrigation, and under outdoor conditions. The respective eluate concentrations were measured and investigated on their environmental relevance with respect to currently allowed threshold values for similar cases and leachate data of previous studies on mineral building materials. The material C^3^ showed a low leaching in all cases and was found to be environmentally compatible. However, outdoor concentrations often exceeded values measured in the laboratory tests and show a significantly different release pattern which leaves the question whether current assessment concepts are applicable for intermittently wetted construction products. This question becomes more relevant in case of more critical emissions.

In order to evaluate this problem, this work examines different approaches on their suitability and adaptability on the emission prediction of C^3^. This way an attempt was made to calculate the long-time emissions of the examined material, and to relate the DSLT leaching data to the actual irrigation data. As not many concepts regarding the emission prediction of irrigated concrete exist, the conventional German method for building components in permanent water contact [15,16], the approach of the Netherlands, applying to intermittent wetted cementitious materials [27], and the modelling program COMLEAM [31,32], designed for the release of organic substances from construction products, are tested.

Subsequently, as for the evaluation or development of an appropriate assessment method, the leaching mechanisms and especially the emission determining factors should be known to provide for a short term test or a transfer model meeting real conditions, a comprehensive data analysis is conducted.

## 2. Methods

### 2.1. Experimental Setup

To determine the leaching behavior of C^3^ a DSLT according to [13], a laboratory irrigation, and outdoor exposure were conducted on test specimens with different sizes, conditions and concrete covering. Samples were collected from the respective runoff or leachates and analyzed on several heavy metal and trace elements concentrations.

The detailed experimental setups and methods used for data acquisition are described in [25], the measured data are to be found in the electronical annex of [25].

### 2.2. Cumulative Release

Following [13] and based on the determined concentrations of [25], the emissions and the cumulated release were calculated using Equations (1) and (2). For the outdoor experiments the concentrations of the blind test were subtracted from the concentrations of the eluates from the test specimens to compensate for the background concentrations in the rainwater and potential contamination from deposited particles.

(1) $$r_{i}=\frac{V_{i}}{A}\cdot c_{i}$$

(2) $$R_{n}=\overset{n}{\underset{i=1}{\sum }}r_{i}$$

where r_i_ = release during the interval i in mg/m^2^, V_i_ = volume of eluate applied in interval i in L, A = surface of the sample in m^2^, c_i_ = concentration of element in the eluate of interval i in mg/L, R_n_ = cumulative release including the intervals 1 to n in mg/m^2^.

### 2.3. Contact Time

The contact time for the laboratory experiments was determined by the duration of the test (in case of the DSLT) respectively the time span of each irrigation period. For outdoor exposure the weather data from the nearby weather station “Aachen Hörn” [33,34], positioned on a roof within 800 m distance from the testing site, with 10 min resolution were used. All intervals with recorded precipitation were counted and assumed as continuously wet. This approach was chosen based on the high data resolution, which allows minor deviations per increment. Moreover, the assumption was made that the error of semi-dry intervals accounted for as totally wet is averaging with the error of test specimens remaining wet for a certain period of time after actual precipitation.

### 2.4. Outlier Identification

**Figure 1:**
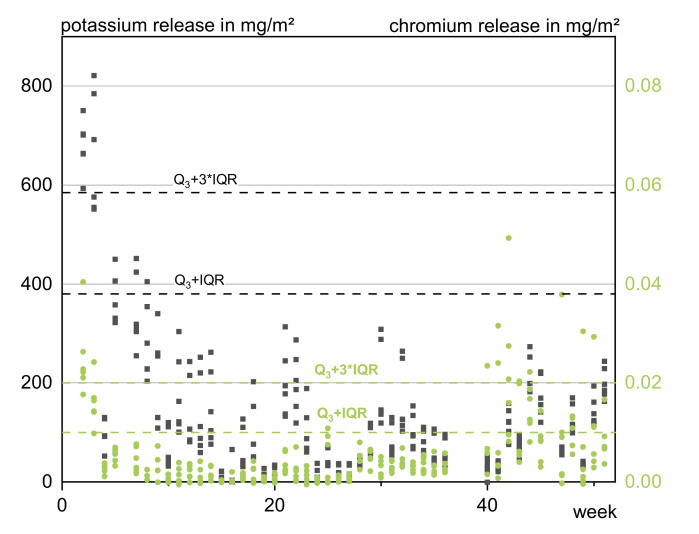
Scatter plot of the incremental releases of potassium (black) and chromium (green) over the testing period of 1a.

The complex interaction of the influencing factors resulted in a wide data distribution, exemplarily pictured in Figure 1 for potassium and chromium. It was therefore difficult to define criteria for a statistical outlier model.

The usually robust outlier test build on a multiple of the interquartile range (IQR) [35] identified the data of practically all first rain events and also plausible values after drying periods as outliers when using Equation (3).

Q_1_ − 3 × IQR > x > Q_3_ + 3 × IQR (3)

where Q_1_ = first quartile, 25% of the data; Q_3_ = third quartile, 75% of the data; and IQR = interquartile range, defined as Q_3_ − Q_1_.

As a consequence the obvious outliers (e.g., an incremental molybdenum release of 2 mg/m^2^ in comparison to an average of 0.037 mg/m^2^) were sorted out manually by assessment of the incremental release values (calculated after 2.2). The release instead of the concentrations was used to eliminate the impact of background concentrations and the runoff amount on the measured values. Besides the absolute value of a specific incremental release, the environmental factors and replicate samples were considered to estimate the plausibility of the value. In conclusion 1.02% of the data points were screened out or, if applicable, replaced by the value of the replicate. Replacement was favored before discarding because missing values lead to constant instead of increasing release. In situations of heavy rainfall even one missing value may cause improbable release developments and high deviations in the final cumulative release.

### 2.5. Transfer Functions and Modelling

#### 2.5.1. Approach of the Soil Quality Decree

The Dutch soil quality decree defines a transfer function (see Equation (4)) to predict the long term leaching behavior by using data obtained by the DSLT. Building components are categorized in two categories: A and B of which B is for irrigated, partially wet components and defined as wetted during 10% of exposure time.

(4) $$I_{\mathrm{soil}}{=E}_{\mathrm{material}}{=E}_{64d}\cdot f_{\mathrm{ext}-V}\left({h,\;x\%,\;D}_{e}\right)\cdot f_{\mathrm{temp}}$$

where I_soil_ = immission into the ground in one year, respectively 100 years in mg/m^2^, E_material_ = emission from the building component in one year respectively 100 years in mg/m^2^, E_64d_ = result of the DSLT after 64 days in mg/m^2^, f_ext-V_ (h, x%, D_e_) = extrapolation factor from 64 days to years, f_temp_ = temperature correction factor from laboratory to outdoor.

The soil quality decree sets the factor f_temp_ to 0.7. The factor f_ext-V_ is calculated considering the thickness, the wetting time and the diffusion coefficient of common building materials. It was agreed on using f_ext-V_ = 5 in order to calculate the 100 year cumulated emission and f_ext-V_ = 0.8 to determine the one year cumulated emission of irrigated construction elements [27].

This function was used to predict the 1 year and 100 year cumulated emissions based on the data obtained from the DSLT.

#### 2.5.2. Modelling with the Software COMLEAM

The non-commercial software COMLEAM (version 2.0) [31], developed and provided by the HSR, University of Applied Sciences Rapperswil (Rapperswil-Jona, Switzerland), and financed by the German Environment Agency (UBA), is a tool to assess the leaching of organic substances from building components exposed to wind and rainfall on a macroscopic scale. As the model has been successfully used for the assessment of the aquatic risk by potential harmful substances [32,36], e.g., biocides and organic additives, the software is tested for its suitability for inorganic elements using the leaching data of this work. It has to be considered, that most likely processes on a microscopic scale determine the release of the investigated elements but since the processes are induced by external factors, an adaption might be possible.

The software allows defining building geometries, weather data, and surface materials to calculate the runoff and resulting emissions from buildings by using customized emission functions. Moreover, the concentration course resulting from the modelled emissions can be calculated for different environmental compartments, e.g., surface water classes. To describe the runoff emission correlation, the different functions have to be provided with coefficients derived from experimental studies.

##### Input Data

The software uses Equation (5), taken from DIN EN ISO 15927:2009-08, to calculate the runoff from provided weather data [32],

(5) $$r_{SR}=\alpha \cdot r^{0.88}\cdot w\cdot \cos\left(\gamma \right)$$

where *r_SR_* = wind driven rain in mm, α = location factor (dimensionless), *r* = amount of precipitation in mm, *w =* wind speed in m/s, γ *=* angle between building exposition and wind direction in °.

Since the total amount of rain that hit the laboratory test specimens was directly measured, no wind driven rain was calculated.

For outdoor simulations the hourly averages for wind direction, wind speed and precipitation of the actual weather data of the weather station Aachen Hörn [33,34] and the runoff coefficient for uncoated concrete of 85% were used. Because of the exposed position with low obstruction possibilities and the comparably small test specimens size, the location factor α was set to 1.

**Figure 2:**
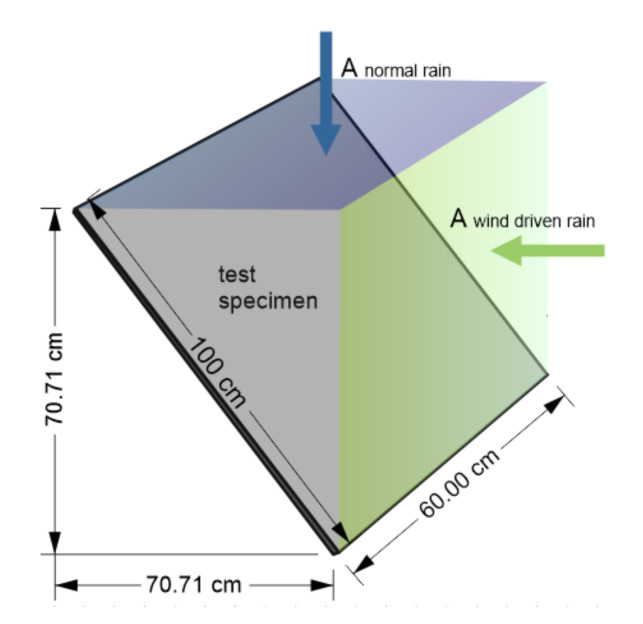
Scheme of the fictitious building surface used for wind driven rain calculations.

Regarding the geometry data COMLEAM does only distinguish between facades (90°) and horizontal components (0°) to calculate the amount of wind driven rain [31]. To test the sensitivity and take the 45° ground angle of the test specimens into account, additional calculations were made with the two imaginary building components pictured schematically in Figure 2. The surface for normal rain and the surface for wind driven rain were defined as two parts of one building in the geometries section. The geometry data used for the simulations are summarized in Table 1.

##### Emission Functions

The runoff to emission correlation in COMLEAM can be described by five different functions, which were chosen on the basis of fitting existing mathematical descriptions with the prerequisite of a constantly decreasing slope. These functions are not necessarily describing the real physical processes. They are called: “Logarithmic function”, “emission function for limited growth”, “Langmuir emission function and Michaelis–Menten emission function”, “double logarithmic emission function”, and “diffusion controlled emission function” [32]. These functions were adjusted and parameterized to match the emission processes of irrigated building components. Another option to describe the runoff to emission correlation in COMLEAM is, to implement a dataset of measured runoff and corresponding cumulated emissions.

The logarithmic emission function (Equation (6)) turned out to describe the emission course of organics from irrigated construction elements in the most appropriate way [32]. Since inorganic trace element emissions from concrete with permanent water contact are mainly solubility and diffusion controlled, both functions and a measured dataset are tested in this work to model the laboratory irrigation experiment. Equation (7) shows the function used for diffusion controlled release in COMLEAM. It becomes apparent, that this modified function does not describe a real diffusion controlled process, as it considers only the square root of the amount of water applied, but no time factor, which is a basic parameter of diffusion.

(6) $$E_{\mathrm{cum}}=a\cdot \ln(1+b\cdot q_{c,\mathrm{cum}})$$

(7) $$E_{\mathrm{cum}}=k\cdot \sqrt{q_{c,\mathrm{cum}}}$$

where E_cum_ = cumulated release in mg/m^2^, q_c,cum_ = cumulated runoff in L, a = proportional factor “characteristic substance percentage” (dimensionless), b = proportional factor (not defined) in m^2^/L and k = “diffusion coefficient” in m/$\sqrt{L}$.

The following input parameters were used for this work; the parameters listed in Table 2 were derived from the laboratory irrigation data by regression with the least square method.

For the implementation of full datasets the runoff–emission correlations from the DSLT, the laboratory irrigation and the outdoor data were used.

### 2.6. Spearman Correlation

To evaluate the correlation between the weather data and the observed substance emissions as well as the mutual correlations between the substances, nonparametric correlation estimations on monotonic relationship after Charles Spearman [37] were conducted using the software SPSS version 25.0 and Origin 2019b. Due to the lack of normal distribution and the wide data range a parametric test (e.g., Pearson) was no option.

The Spearman correlation coefficient (r_s_) is based on the ranked values for each variable and thereby considering only the order instead of the total value of the raw data. The coefficient r_s_ is basically calculated after Equation (8), ties are considered in an extended equation by the number of their incidence [38].

(8) $$r_{s}=1-\frac{6\cdot \sum_{i=1}^{n}\;{\left(r_{i}-s_{i}\right)}^{2}}{n^{3}-n}$$

where r_s_ = Spearman rank coefficient, r_i_ = rank of variable X of data pair i; s_i_ rank of variable Y of data pair i; and n = number of data pairs.

The test is considered as robust against wide ranges and outliers [39,40]. Nevertheless, the impact of the first months of exposure (see also Section 2.3) on the correlation factor r_s_ was tested by running a second calculation leaving the first four weeks apart. An improved Spearman correlation value (r_s_) for 13% of the data was observed but 49% were downgraded. The influence on strong correlations was expectedly low so the full data set was used for further assessment.

To describe the strength of the correlation factor r_s_ a rough classification was done in reference to Kendall [38] and Cohen [41]. In this work r_s_ is referred to as weak for |r_s_| ≤ 0.25; moderate for 0.25 < |r_s_| ≤ 0.50; strong for 0.50 < |r_s_| ≤ 0.75 and very strong for 0.75 < |r_s_| ≤ 1.00. The *p*-value was also calculated and the significance level set to *p* = 0.05.

To recognize relationships other than monotonic (e.g., parabolic), scatterplots were created additionally and inspected on their course.

### 2.7. Multiple Regression

To calculate linear multiple regressions Minitab^®^ 19 Statistical Software was used. Key assumptions for this kind of regressions are:

- A linear relationship between input parameters and the outcome variable;
- No multicollinearity of input variables (The software excludes strongly correlating variables by regression of one predictor on another one. Moreover, collinear input parameters were partially excluded by knowledge based selection in advance.);
- and homoscedasticity of residuals, ratable by the software’s residual plots.

The interactions between the twelve possibly determining, partially correlating, variables:

- Time of exposure in days (t_ex_);
- contact time in hours (t_con_);
- air temperature in °C (T);
- normal rain in mm (NR);
- wind driven rain in mm (WDR);
- total rain in mm (TR);
- rain intensity in mm/h (I);
- runoff in L/m^2^ (runoff);
- wind speed in m/s (v);
- wind direction in ° (α);
- air humidity in % (RH); and
- rain water pH/background concentration.

As well as their respective contribution to the emission value were examined. To take different slopes into account also transformed data (e.g., logarithmized or to the power of −1) were used.

Depending on their integrity, *n* = 302 to 334 datasets were used to fit a function using stepwise backward elimination. In doing so, quadratic equations and terms with twofold interactions between the parameters were allowed. The elimination method starts calculating with all potential terms in the model and removes the least significant terms. The α value for removal was set to 0.1 in the first step. Terms with *p* < 0.05 or with contributions of lower than 0.01% were directly removed from the models as well. The quality of the models is rated by the distribution of residuals and the models R^2^, adjusted R^2^ and predicted R^2^ (see Table A1). If not stated otherwise the adjusted R^2^ is used in the results and discussions section.

## 3. Results

### 3.1. Assessment of Cumulated Release of C^3^ Using the Concepts for Permanent Water Contact

In [26] the eluate concentrations of irrigated C^3^ were evaluated and found to be uncritical. For a long term assessment, also the cumulative total release has to be considered. The conventional assessment methods for building materials in contact to water in Germany and in the Netherlands are therefore applied to the results of the DSLT and for comparison to the other tests conducted.

Table 3 shows the cumulative releases compared to the threshold values after [16] and to the threshold values for soil and groundwater protection of the Netherlands [42], which both apply to the DSLT results. As the German values are based on the assumption of a direct release into the groundwater and refer to the emission from the material whereas the Dutch consider soil retention and are set as immissions values, the Dutch thresholds are less rigorous and probably more suitable for irrigated construction elements.

It becomes apparent that firstly, most substances except for nickel and arsenic show lower releases during the irrigation cases compared to permanent water contact and secondly, even the German thresholds are not reached by any substance in any test. The element closest to the threshold would be vanadium with a release of 2.18 mg/m^2^ in the DSLT and a threshold of 4.4 mg/m^2^; however this threshold is currently suspended. Next would be antimony with 1.34 mg/m^2^ released compared to a threshold of 5.5 mg/m^2^.

**Figure A1:**
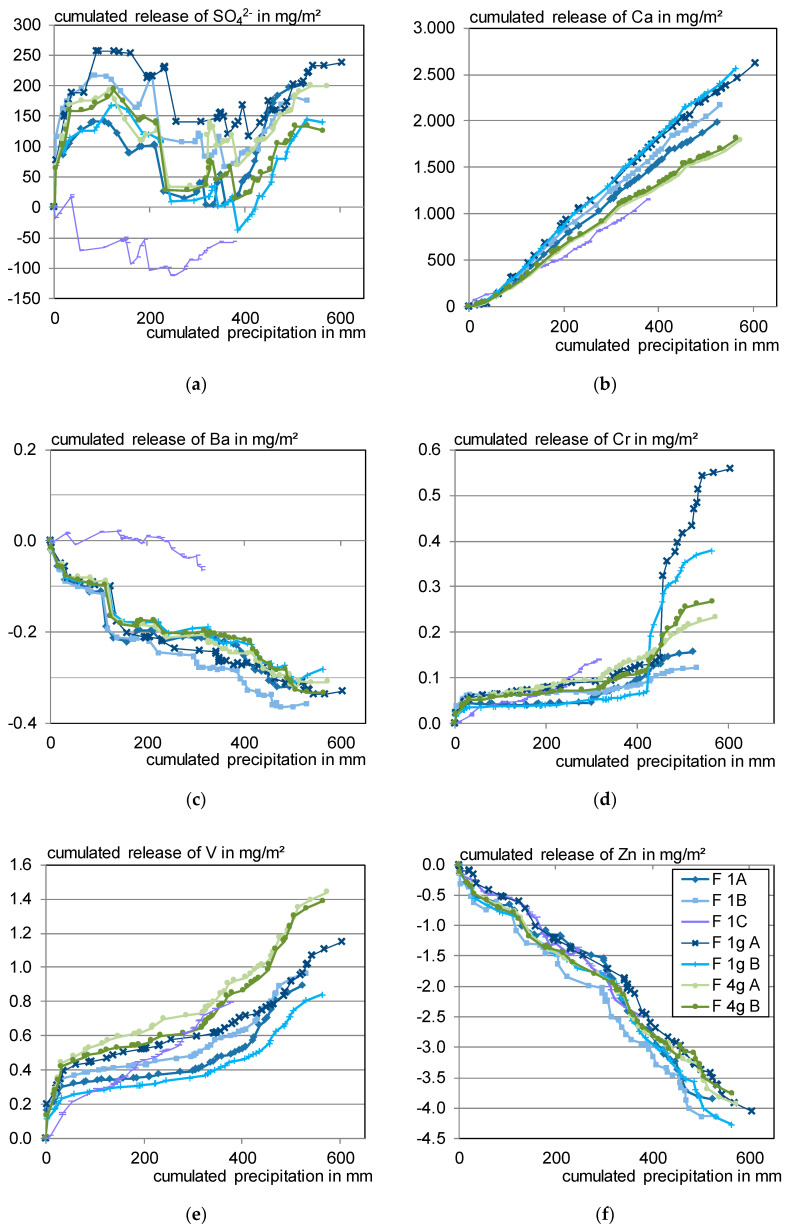
Cumulative release of (**a**) sulfate, (**b**) calcium, (**c**) barium, (**d**) chromium, (**e**) vanadium, and (**f**) zinc. Negative values occur due to adsorption of substances contained in the rain water.

A tendency of the 4-layer, cracked surface specimens towards a higher release can be assumed for sodium, potassium, arsenic, copper, molybdenum, selenium, and vanadium from the outdoor results (see also Figure A1). Nevertheless this is not a significant difference and might also be a result of the multiply cracked specimens and thus an extended surface. This can be confirmed by the lab results from intact surfaces and also the differences in the total release of most substances from the specimens F 1gA with 2–3 cracks and F 1gB with only one crack. An influence from the carbon reinforcement and its SBR coating on the release of heavy metals and trace elements is therefore improbable.

**Figure 3:**
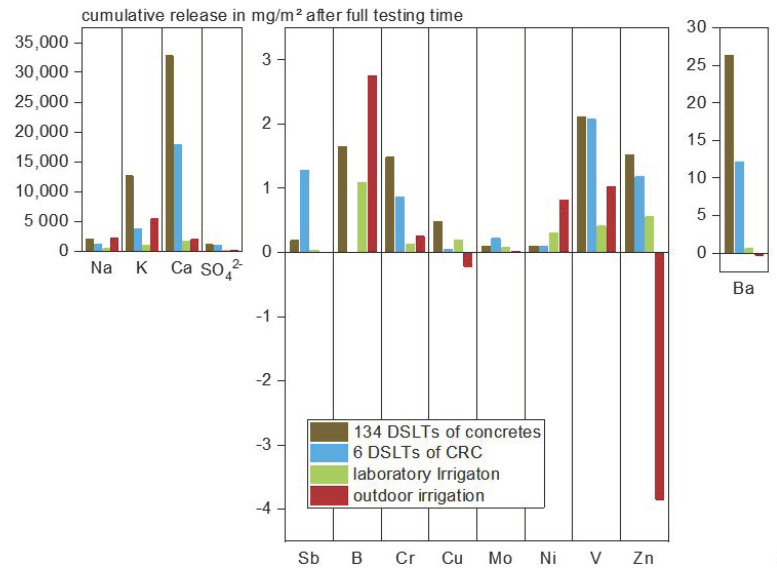
Cumulative release of selected substances from concretes after 64 days of DSLT, 28 days under laboratory irrigation and 365 days of outdoor exposure; comparison of the median of 134 DSLT data sets to C^3^.

Also when looking at the median releases of cementitious materials collected in an in-house database from the Institute for Building Materials Research (134 DSLTs) and comparing it to the average releases determined in this study (see Figure 3), it is revealed, that the overall cumulative release of all substances, except for antimony and molybdenum, is lower than the median release observed in the previous DSLTs. Since the C^3^ consists of a fine grained concrete with a low water binder ratio and a dense matrix, this is an expected effect. It also shows once more that very likely no matrix-reinforcement interactions are influencing the leaching of heavy metals.

The direct comparison of the cumulative releases to the threshold values and to similar materials allows, in accordance with the findings of [25], a positive evaluation of the emission behavior of C^3^ in terms of leaching.

However, it has to be mentioned that the applied assessment method and therefore the threshold values can only be seen as a benchmark. As the concepts are designed for the case of direct contact between concrete and groundwater, including an immediate dilution, lower allowable emission values may have to be applied on irrigated construction elements. In order to define new limit values the point of compliance has to be agreed on first. If the leachate is considered to infiltrate in a soil compartment and the assessment takes place in a certain soil depth or even in the groundwater, interactions of the leachate with the soil and dilution with pure rainwater or groundwater can be considered as diminishing factors. In that case lower thresholds than the ones of [16] can be expected. These aspects are not focused on further in this paper as it only deals with the prediction of the source term.

### 3.2. Transfer and Modelling

Since no direct correlation between the laboratory results and the outdoor leaching behavior can be determined, subsequent transfer options are investigated on their suitability. The results of the approaches specified in 2.5 are summarized in the following. Due to their consistent but distinct leaching behavior, the elements vanadium and barium were chosen exemplarily for the discussions; results are illustrated using the test specimens and rain intensities of L 1–2 A respectively F 1 A.

#### 3.2.1. COMLEAM

##### Laboratory Irrigation

Using the software COMLEAM with the input data described in chapter 2.5.2, it was possible to reproduce the experimental irrigation data. Consistent with [32], the logarithmic function performed best (see also Figure 6) even compared to the original dataset. Therefore, further modelling was done using this function. The modelled effect on environmental compartments, in which the rainwater run-off infiltrates, is not further analyzed in this paper.

The software was not applicable to use the compiled laboratory data of this work for further prognosis and assessment. The DSLT was developed to match diffusion controlled processes, therefore the same amount of water is applied in different time steps. Since all available functions, even the “diffusion function”, are neglecting the factor time, it is not possible to describe the runoff–emission relation well.

**Figure 4:**
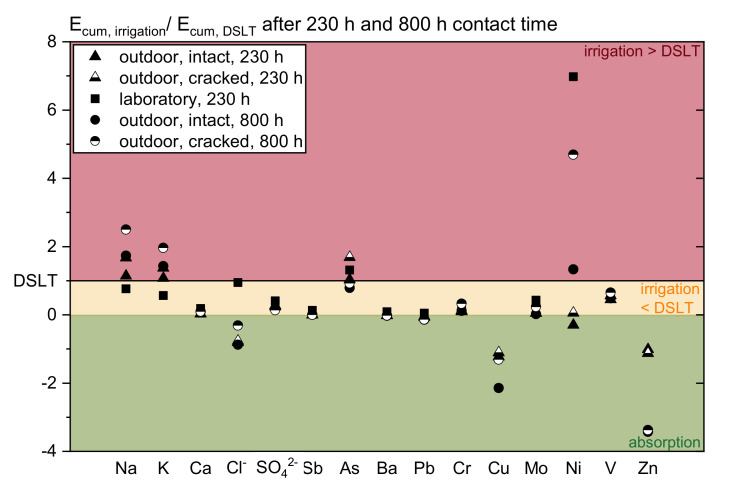
Relation of the averaged cumulated release of the irrigation scenarios related to the DSLT after 230 and 800 h of contact time; 800 h ≙ precipitation time during one year.

**Figure 5:**
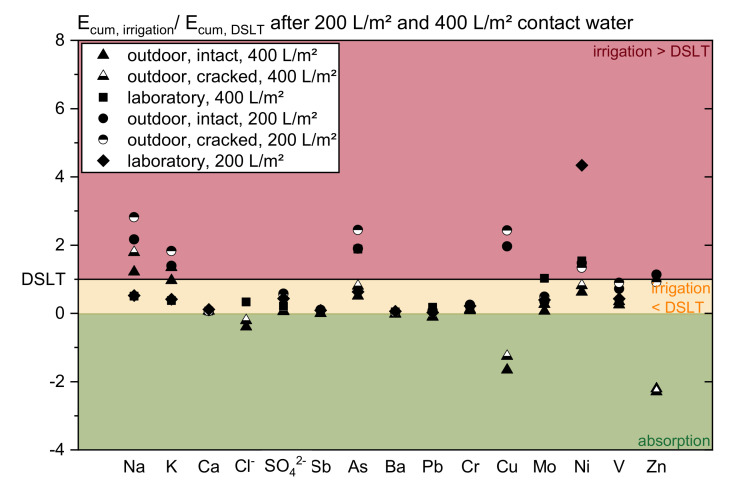
Relation of the averaged cumulated release of the irrigation scenarios related to the DSLT after a runoff of 200 and 400 L/m^2^.

To illustrate the problem of the emission data related to contact time or runoff, Figure 4 and Figure 5 show the relation of the averaged cumulated releases of both irrigation investigations of all specimens’ types to the DSLT. The evaluation is done at specific contact times (230 and 800 h) and specific water amounts (200 and 400 L/m^2^).

The different progresses of the leaching and the significance of the influencing factors become visible. In many cases (for Na, SO_4_^2−^, As, Cu, Mo, V, and Zn) the emissions at the considered contact times show a slightly better agreement than at the two cumulated amounts of water applied. However, a systematic relation between the tests cannot be observed. Depending on the substance the ratios vary widely. It is clearly revealed that the amount of water and the contact time are not the only main factors determining the release. Hence, the complexity of concrete leaching cannot be described in one function.

**Figure 6:**
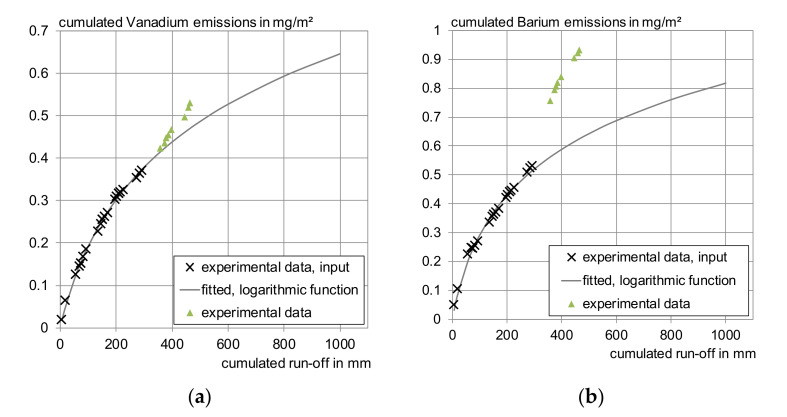
Fitted emission functions compared to experimental data: (**a**) Vanadium, (**b**) barium.

Figure 6 illustrates this problem. The input function derived from experimental laboratory irrigation data of four weeks is compared to further experimental data. The second irrigation sequence was carried out six month after the end of the first irrigation period; during the storage time the test specimens were exposed to the laboratory environment (20 ± 4 °C, 60 ± 15% RH). It is visible, for barium more strongly than for vanadium, that the logarithmic function based on the first two sequences describes the first four weeks of irrigation well but cannot be used as a prognosis for the last two weeks. Even at low concentrations, as measured in this work, and at the controlled ambient conditions in the laboratory, longer phases in which the sample dries show an unneglectable influence. Since the leaching behavior changes over time, due to an altering microstructure and decreasing pH due to carbonation, it might be helpful to include other functions derived from either experimental data and/or geochemical modelling in this simulation, in case that a precise modelling of inorganic substances is targeted.

##### Field Tests

**Figure 7:**
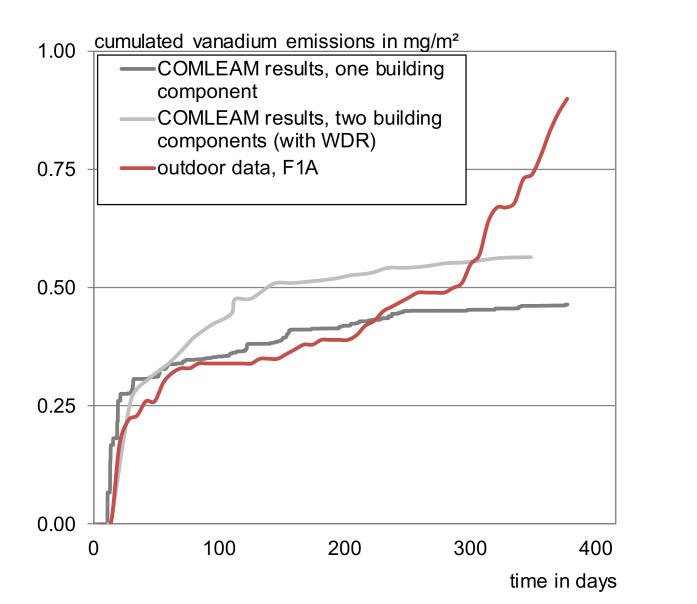
Cumulated emissions of vanadium from F 1 A after one year of exposure; actual release compared to COMLEAM calculations.

The outdoor experiment was modelled for the sample F 1A. The log-function describes a continuously decreasing slope so that it could obviously not picture the process of the outdoor simulation (compare Figure 7 and Figure A1), modelling was therefore conducted using the full dataset of the runoff–emission correlation, the actual weather data, and the runoff coefficient of 85%. In this case, the program calculates the run-off from the weather data and shows the corresponding emissions. As for the laboratory experiments it was possible to picture the actual process. The simulation led to a runoff of 567 L/m^2^ (collected: 524 L/m^2^) and cumulated vanadium emissions of 0.96 mg/m^2^ (measured: 0.9 mg/m^2^) after one year.

Predictions for the even more complex outdoor leaching behavior of concrete cannot be described by the software yet. Figure 7 exemplarily shows the approach of calculating the second half of the testing year for vanadium by implementing the first half as input data and using the same factors as the reproduction simulation. With 0.48 mg/m^2^ only approximately half of the amount released in the outdoor experiment is predicted as cumulated release after one year.

#### 3.2.2. Transfer Functions

For constantly wet construction elements the Dutch approach [27] provides formulae to extrapolate cumulative emission data from the DSLT to outdoor exposure after certain time periods. For the case of partially moistened elements [27] it suggests the factors 2.4 (1 year) and 15 (100 years) using Equation (4) described in paragraph 2.5.1. This turns out to just be a factor of 1/3 to calculate from constantly wet to irrigated components.

CEN/TS 16637-2, Annex b.7.4 [13] provides a formula based on the diffusion function to estimate cumulative emissions for permanently wet components on the basis of the DSLT results.

**Figure 8:**
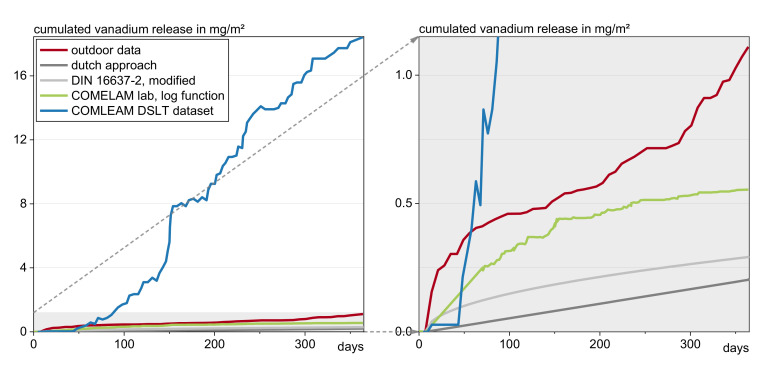
Prognosis of the cumulated vanadium release after 1 year of irrigation, calculated with different approaches.

Figure 8 shows the different prognoses for vanadium by using:

- The Dutch approach, Equation (4), for intermittent moistened materials;
- the extrapolation after CEN/TS 16637-2 for permanently wet materials and applying the factor of 1/3 from the Dutch approach on this results;
- the COMLEAM calculations with an input dataset of the DSLT; and
- the COMLEAM calculations with an input logarithmic function derived from lab irrigation.

The calculation results are compared to the actual outdoor release.

Using the DSLT data as an input for the software COMLEAM leads to a very high overestimation. The data extrapolated from the DSLT by the Dutch and the combination of German/Dutch standards can be seen as similar after one year, but the actual emissions are underestimated by a factor of 4.

It is revealed that every calculation method provides a different estimation. Even the same method delivers different tendencies for different substances.

Table 4 shows the prognoses after the Dutch approach [27] leading to extremely inconsistent over- and underestimations.

An assessment concept will have to consider more environmental factors and also distinguish between the substance characteristics.

## 4. Discussion

All known available methods to assess irrigated construction elements are based on laboratory tests and only refer to the factors time and/or amount of water applied. They are not yet applicable to estimate and therefore assess the emissions of irrigated concrete.

### 4.1. Leaching Patterns in the Field Experiments

Different influencing factors that are not considered in laboratory tests lead to different outdoor leaching patterns. Characteristic release graphs of the substances of the same leaching behavior are grouped and summarized in Table 5. For comparison the actual release curves of sulfate, calcium, barium, chromium, vanadium, and zinc over the testing period of one year are exemplarily shown in Appendix A, Figure A1.

It is noticeable that the oxyanion forming metals form one pattern group (4a) which would point at a pH-dependent leaching or a change in the chemical structure, e.g., decomposition of cement hydrates such as ettringite. As the cations show a similar pattern (4b) it is assumed that external factors, determining the transport mechanisms, are responsible for a part of the emissions.

In [6] Scherer observed similar patterns for the leaching of the elements boron and antimony from renders as found for arsenic, boron, chromium, and vanadium. Contrary to the findings of this work, vanadium and chromium were assorted to a more linear leaching course. However, the slope changes observed for the leaching of C^3^ can be, especially in the case of chromium, identified for the mortars of [6] as well (see pp. 104–111 of [6]). The leaching behavior therefore seems reproducible for cementitious materials. In [6] it was assumed, that particle deposition or surface damage by weather, for example hail, led to the increase phases of group 4 emissions. Both can be excluded as dominant factors for this work, as, apart from the mentioned deposits, the blind test showed no irregularities at the respective events, hail was not observed and frost (at around 180–200 mm cumulative runoff) had no significant influence on the subsequent release.

### 4.2. Influencing Factors on Outdoor Leaching

#### 4.2.1. Spearman Correlation

**Figure 9:**
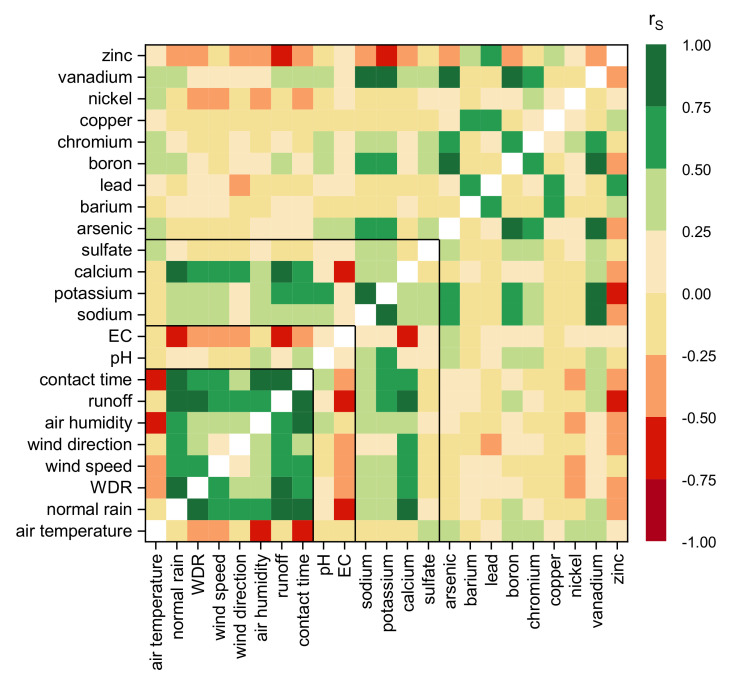
Spearman correlation of weather parameters and substance leaching.

To calculate the effects on the outdoor leaching behavior, the main effects on the release have to be considered. The data of this work were examined concerning their correlation between weather data and emissions by calculating the Spearman correlation coefficient. The results are presented as a heat map in Figure 9, where strong positive correlations are pictured in dark green and strong negative correlations in dark red. All correlations |r_s_| > 0.3 are significant on a level of *p* = 0.05. Seven insignificant moderate correlations (0.25 < |r_s_| < 0.3) were calculated: pH—contact time, pH—chromium release, air temperature—sulfate release, EC—arsenic release, normal rain—boron release, vanadium release—calcium release, and zinc release—arsenic release.

**Figure 10:**
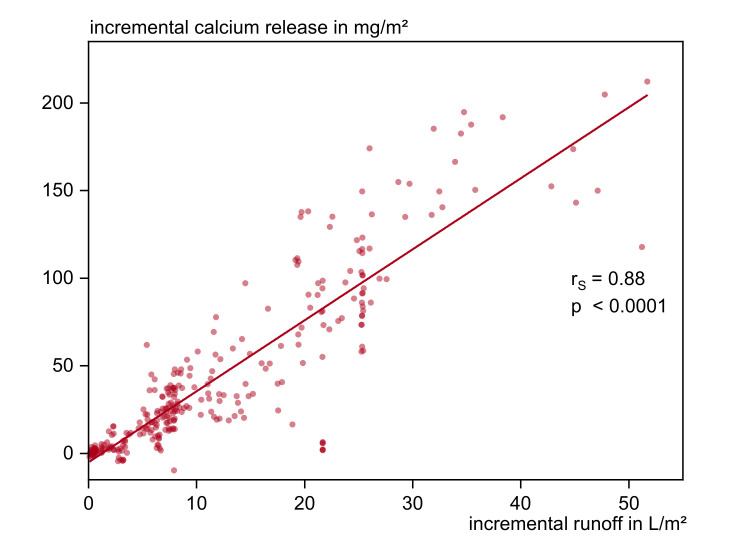
Incremental calcium leaching related to amount of runoff.

It is revealed that calcium and zinc are the only substances showing direct, strong correlations to single weather parameters. Figure 10 shows that under outdoor conditions calcium leaching can be seen as exclusively dependent on the amount of water applied. The emissions correlate very strong to the amount of normal rain/run-off (r_s_ = 0.84, *p* < 0.0001 and r_s_ = 0.88, *p* < 0.0001) and strong for wind driven rain (r_s_ = 0.63, *p* < 0.0001) respectively weather parameters influencing WDR. Zinc uptake correlates strong with runoff (r_s_ = −0.51, *p* < 0.0001) and moderate with contact time (rs = −0.43, *p* < 0.0001).

Air temperature and air humidity show a strong mutual correlation but different impact on the substances. Mainly diffusion controlled heavy metals are more sensitive to temperature. Some moderate correlations to air temperature but not to humidity were determined for the leached substances sulfate (r_s_ = 0.25), vanadium (r_s_ = 0.27), nickel (r_s_ = 0.37), chromium (r_s_ = 0.50), boron (r_s_ = 0.40), and arsenic (r_s_ = 0.33) while low air humidity seems to accelerate the capillary transport of the more soluble substances like sodium, potassium and calcium independently from temperature.

Contrary to the other heavy metals boron and vanadium additionally correlate moderately (r_s_ = 0.30 and 0.40) to the amount of runoff.

**Figure 11:**
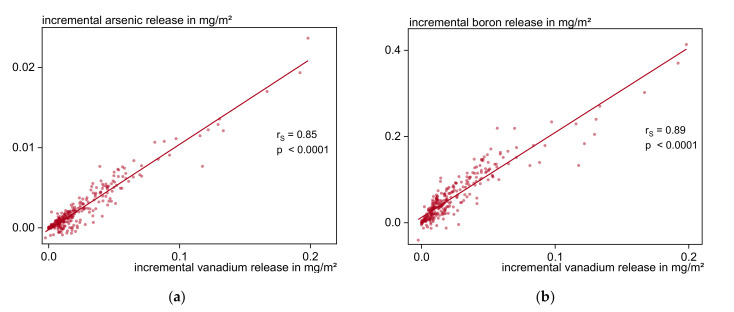
Incremental release of (**a**) arsenic and (**b**) boron in relation to vanadium release (group 4 of Table 5).

Furthermore, the pattern groups formed and presented in Table 5 are verified. Figure 11 underlines that very strong (r_S_ > 0.75) to strong (r_S_ > 0.5) linear correlations occur between the substances of group 4 with ratios of 10:1 for vanadium to chromium and arsenic, and 1:2 for vanadium to boron. Because of its chemical similarity and the present moderate correlations to sodium (r_s_ = 0.35), potassium (r_s_ = 0.31), arsenic (r_s_ = 0.41), boron (r_s_ = 0.50), chromium (r_s_ = 0.34), and vanadium (r_s_ = 0.35) sulfate is most probably a part of this group as well.

All correlations determined for the grouped substances are most likely not causal and therefore suggesting that the release of the groups is determined by the same parameters. The grouping probably could be used to divide the observed parameters into three to four transfer groups for an assessment concept.

#### 4.2.2. Influence of Dry Phases

Since the correlation calculation was not able to straightly prove single influencing parameters on most substances’ leaching behavior, the influence of dry phases, which is known to be significant (compare also Figure 6) is examined by qualitatively relating the release curves to weather events.

**Figure 12:**
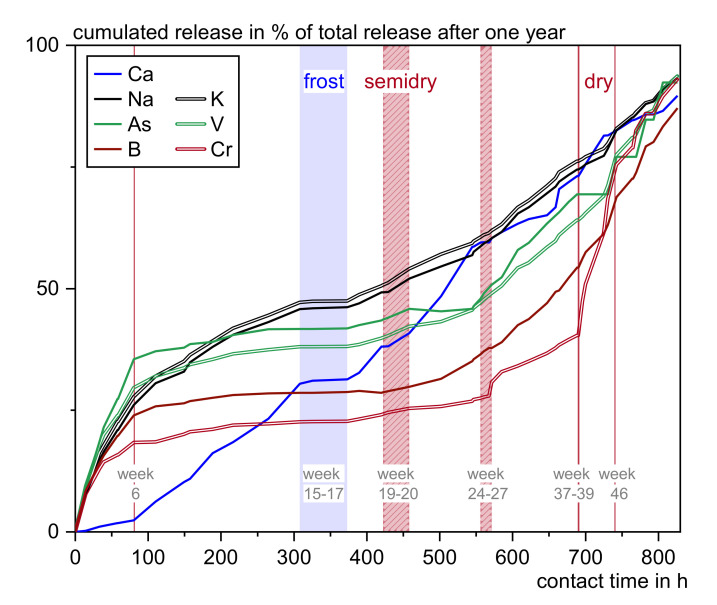
Cumulated release of selected elements as a percentage of their total release related to contact time and weather.

Figure 12 shows the cumulated release of calcium and the elements of group No. 4 as a percentage of their total release related to contact time and weather. The first change in the leaching process for all substances correlates also to the first drying phase in week 6, suggesting a physical change in the pore structure. Most likely this traces back to an accelerated carbonation as carbon dioxide can enter the concrete better and dissolves in the pore water by a partial drying of water filled pores. This may cause a densification of the cement matrix or at least a covering of the respective, substance incorporating, phases. This theory is supported by the observed pH drop from ~pH 9 to pH 7 ± 0.5, increased calcium leaching, and decreased leaching of sulfate, arsenic, boron, chromium, molybdenum, selenium, and vanadium. Lead is absorbed to a higher extent [25], probably due to elevated background concentration, and might be replacing calcium [43]. Only sodium and potassium as easily soluble constituents are only indirectly influenced by carbonation, due to changes in the pore structure.

The next obvious changes can be observed at week 20 after two and from week 28 after four weeks of low precipitation and therefore phases of probably half wetted concrete. After week 20 calcium, sodium, and potassium stay unaffected while arsenic, boron, chromium, and vanadium releases increase. Since this effect is only visible shortly after the semidry phase, it is attributed to capillary transport. The next semidry phase results in a further increase of the anionic substances pictured in Figure 12. An explanation for this phenomenon would be an advanced carbonation process, leading to a degradation of ettringite and C–S–H and therefore the release of incorporated substances [44].

The total dry phases rise heavy metal leaching but show no effect on sodium and potassium. Calcium is leached but does not seem to be influenced immediately by the drying cycles.

As a conclusion to the simultaneous changes in the leaching process, it can be assumed, that matrix change is the prevalent factor affecting the leaching behavior of many investigated substances, often leading to the characteristic curves No. 2 and 4. It is assumed that heavy changes occur especially after semidry phases as a result of accelerated carbonation and precipitation of dissolved substances and after dry phases as a result of increased capillary transport by the greater humidity gradient.

#### 4.2.3. Multiple Regressions

The influence of interactions of the aging matrix and weather conditions on the substance leaching are not to be shown by the spearman correlation and can be assumed but not quantified by interpreting the release curves. A multiple regression was therefore conducted to formulate a model which can describe mutual interactions and quantify the observed emission behavior.

Six input parameters were found to be decisive for most of the examined substances: Time of exposure (t_exp_), contact time (t_con_), air temperature (T), air humidity (RH), and runoff; for the leachate pH and zinc uptake additionally the respective background concentrations. Using these factors, it was possible to calculate the incremental releases of all parameters with correlation factors of R^2^ > 0.7. A consideration of the individual runoffs mostly led to an improvement of ~2%, showing a better adaption to the spread between the respective test specimens. A categorization into the three categories following a dry (Y), semidry (S), and wet phase (N) led to an estimation improvement of 0.1 to maximum 4%; however, at a disproportionate increase of function complexity and partially seeming to result into overfitting.

**Figure 13:**
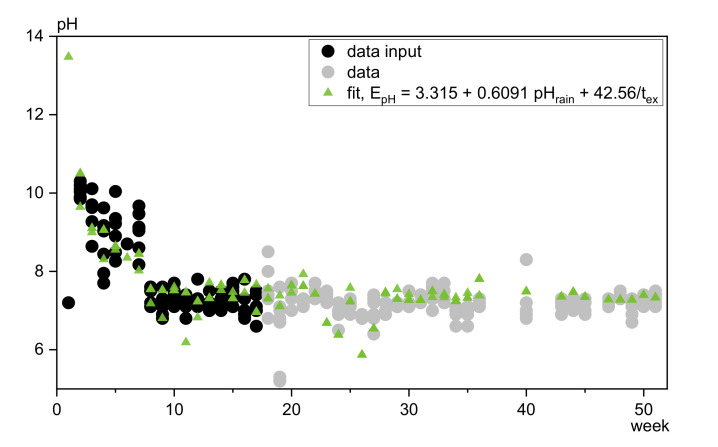
Fit and resulting prognosis of incremental pH values in comparison to original data.

**Figure 14:**
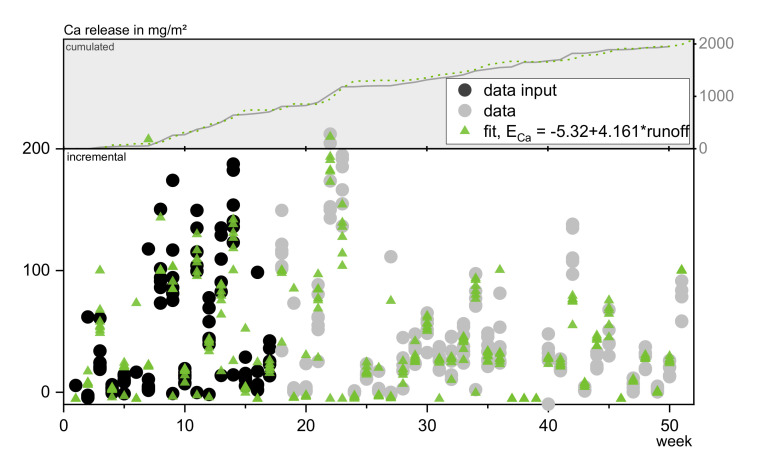
Fit and resulting prognosis of calcium release in comparison to original data.

**Figure 15:**
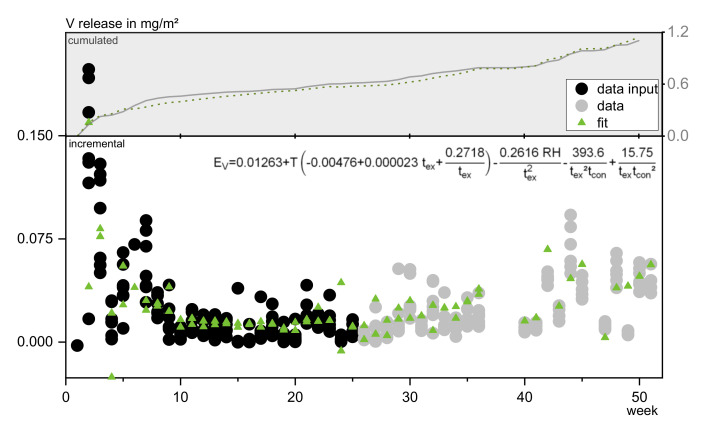
Fit and resulting prognosis of vanadium release in comparison to original data.

**Figure 16:**
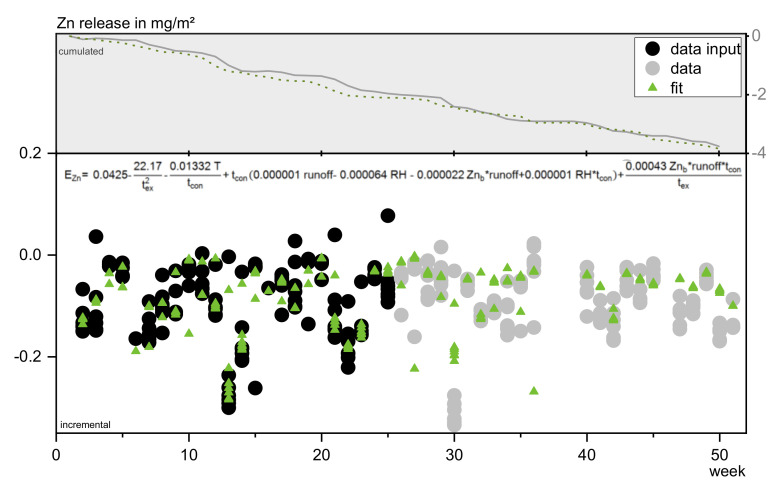
Fit and resulting prognosis of zinc release in comparison to original data.

Figure 13, Figure 14, Figure 15 and Figure 16 show the fitted data for pH, calcium, vanadium, and zinc. A third to half of the collected data was used to fit a function and predict the remaining part of the test period. Table A2 summarizes the contributions of single terms and therefore parameters to the whole model. 

##### pH

As can be concluded from Figure 13, 82% of the measured leachate pH can be described by terms considering the time of exposure (40%) and the background pH (42%) of the rainwater. Including RH or t_con_^2^ into the function will improve the R^2^ by around 2% but does not change the general course. Thus the leachate pH value is mainly resulting from the decreasing materials pH and the background pH of the rain water.

##### Calcium

The leaching of calcium is primary determined by the amount of water applied. A good approximation can therefore already be achieved, as indicated by the Spearman correlation factor, by calculating the calcium emission either as a function of NR (R^2^ = 0.78) or runoff (R^2^ = 0.86). Figure 14 shows the function and fit derived from the first 17 weeks of exposure. Adding a time or temperature factor improves the correlation factor by 1% (t_ex_) to 2% (T, t_con_^2^) and results into a slightly more uniform distribution of residuals.

It becomes visible that the regression function allows a good prediction of the future emissions. However, it has to be mentioned, that the validity has to be tested for more than one concrete material and that the factors are only applicable for the observed case of C^3^. For a long term prognosis t_ex_ will have to be examined for its relevance and contribution again.

##### Group 4, Vanadium

The release of the substances of group 4 can be approximated and predicted by a function derived from half of the data with a combination of the input parameters time of exposure, contact time, air temperature, and air humidity. A factor for the amount of water applied is not immediately considered. As contact time is already covering the amount of rain and runoff to a certain extent, the amount of water shows a minor impact on the release of this substances. Figure 15 exemplarily shows the fit for vanadium. In cases of heavy rainfall (see weeks 6 and 44) the release is underestimated while for low runoffs (weeks 24 and 49) the reduced emissions, probably due to low concentration gradients, cannot be met. However, intensity as an input variable neither was calculated as significant nor led to improved fitting results.

Air temperature and time of exposure are the main influencing parameters, explaining ~70% of the vanadium emissions. This again reinforces the assumption that matrix changes play a major role in the release of group 4 substances, while the diffusion process is subordinate.

As was estimated on the basis of the Spearman correlation results, using a multiple of the vanadium function will depict the main release pattern of arsenic and boron sufficiently, too. Since chromium shows too heavy deflections after the dry period, it could not be modelled well using the available factors.

##### Zinc

Seventy percent of the zinc uptake can be explained using factors based on the rainwater background concentration (Zn_b_) and amount of runoff combined with contact time. Including temperature and RH into the calculation improves the correlation. This is suggesting that zinc uptake in this case is a combination of capillary transport and diffusion into the test specimen, and a temperature dependent chemical process like complexation and incorporation, e.g., as hyroxides or carbonates.

It has to be mentioned, that the previously described, derived functions, especially the coefficients, should thereby not be seen as universal rules and are not describing the underlying physical-chemical process. Pre carbonated samples for instance will certainly show another slope for the exposure time (t_ex_) influence. However, they clearly show the influence of the mentioned parameters on the leaching behavior. Despite the complex interactions it seems possible to predict the leaching behavior of concrete by using the factors responsible for matrix changes and capillary transport instead of modelling the actual process. Considering the uncertainties of an outdoor experiment, the overall low emissions, and possible contaminations or analytical errors, an adequately precise fit was achieved for the examined indicators.

## 5. Summary and Conclusions

- Investigations on the inorganic leaching behavior of carbon textile reinforced concrete confirmed the findings of Part 1 of this study: No environmentally harmful leaching was observed.
- Different approaches were tested to predict outdoor leaching behavior from laboratory data. Calculation models provided by Dutch and German standards mostly underestimated the total release. The tested modelling software COMLEAM does not suit the difficult case of heavy metal and trace elements leaching from cementitious materials, as it is designed for prediction on a macroscopic scale. A different modelling concept is needed for inorganics released from concrete.
- The investigated elements were divided into four groups, characterized by their respective leaching pattern formed through external factors. The influencing factors were determined using a Spearman correlation calculation, whereby most substances show only moderate correlations to single weather parameters.
- The influence of combined weather conditions was calculated. Considering six external factors is sufficient to describe and predict the leaching processes phenomenologically. The main leaching mechanisms (solution and diffusion) remain important but are significantly superimposed by outdoor influences with different impact on the particular substances.
- More research is necessary to develop a matching concept on transfer functions for irrigated building components. For an improved transferability to other cementitious materials, the underlying physicochemical processes should be identified, e.g., using geochemical modelling. The findings of this work concerning pattern groups and influencing parameters are providing a foundation for further assessment method development and definition of physicochemical relations.

## Figures and Tables

**Table 1:** Geometry Data used for COMLEAM.

Scenario	Object ID	Building ID	Facade					Mineral 1 (Concrete) ID
			Width [m]	Height [m]	Area [m^2^]	Exposition [°]	Ground Angle [°]	
Laboratory	1	1	0.30	0.40	0.12	270	45	106
Outdoor	1	1	0.60	1.00	0.60		45	
Outdoor-split	1	1	0.60	0.71	0.42		90	
	2	1	0.71	0.60	0.42		0	

**Table 2:** Emission function input parameters for COMLEAM derived from the laboratory irrigation data.

Element	Logarithmic Function Parameters		Diffusion Parameter
	a	b in m^2^/L	$k\;\mathrm{in}\;m/\sqrt{L}$
Ba	0.271	0.0194	0.0299
V	0.261	0.0109	0.0208

**Table 3:** Maximum cumulative release of substances after 64 days (Dynamic Surface Leaching Test (DSLT)), 28 days (laboratory irrigation) and 365 days (outdoor exposure) of testing in comparison to groundwater protection values after [16] and [42], (green: <1% of German threshold, blue: >15% of German threshold).

Substance	DSLT [mg/m^2^]	Laboratory [mg/m^2^]	Outdoor [mg/m^2^]	Threshold D [mg/m^2^]	Threshold NL [mg/m^2^]
SO_4_^2^^−^	1170	393	233	264,495	165,000
Sb	1.34	0.049	-	5.5	8.7
Ba	17.5	0.869	−0.27	375	1500
Cr	1.16	0.371	0.53	7.7	120
Cu	0.209	0.271	−0.10	15.4	98
Mo	0.324	0.083	0.10	38.6	144
Ni	0.204	0.816	3.64	15.4	81
V	2.18	0.750	1.43	4.4 ^1^	320
Zn	1.23	0.829	−3.61	63.9	800

^1^ Currently suspended.

**Table 4:** Emission prognosis after the Dutch soil quality decree [27] compared to measured emission values.

Substance	Substance Release After 1a in mg/m^2^		Deviation from the Measured Value in %
	Calculated After [28]	Outdoor	
Na	650	2578	−297
K	2086	6393	−207
Ca	14328	2157	85
Cl^−^	30.4	−163	638
SO_4_^2−^	669	180	73
As	0.0395	0.108	−174
Ba	9.03	−0.286	103
Pb	0.482	−0.240	150
Cr	0.631	0.288	54
Cu	0.176	−0.188	207
Mo	0.191	0.0573	70
Ni	0.159	0.511	−221
V	1.27	1.11	12
Zn	0.651	−3.99	712

**Table 5:** Categorization of leaching patterns, outdoor testing compared to the blank.

Sub-Stance	Blank (Glass Plate)		Emission from C^3^	
	Development of Concentrations	Cumulated Amount in mg/m^2^ after One Year	Cumulated Release in mg/m^2^ after One Year	Schematic Release Graph
Ca	Independent from season and weather conditions	220	2157	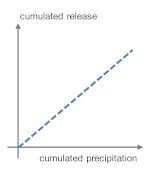 1
Cl^−^	Unstable, consistently in the range of eluate concentrations	870	−163	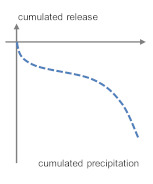 2
Ba	Independent from season and weather conditions	1.11	−0.286	
Pb	Higher concentrations after dry phases, probably due to particle deposit	0.464	−0.240	
Zn		6.09	−3.99	
Cu		0.910	−0.188	
SO_4_^2^^−^	Unstable	609	180	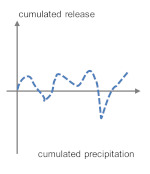 3 *^2^
Sb	Independent from season and weather conditions, but consistently in the range of eluate concentrations	0.081	0.000	
Mo *^1^		0.227	0.213	
B	Independent from season and weather conditions	1.87	3.21	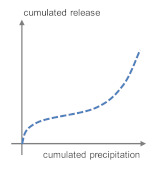 4a
As		0.071	0.108	
Cr	Stable, slight increase from march to august	0.130	0.288	
V		0.143	1.11	
Na	Independent from season and weather conditions	597	2578	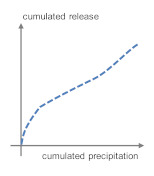 4b
K		71.6	6393	

*^1^ Molybdenum shows a tendency to graph 4. *^2^ Very low emission to blank ratio: High impact of allocation, possible contaminations or analytical errors.

## Appendix B

**Table A1:** Comparison of fit qualities for predictor variations.

Sub-Stance	R^2^ in %	R^2^_adjusted_ in %	R^2^_predicted_ in %
pH	81.76	81.38	80.30
Ca	85.76	85.71	85.42
V	75.20	74.50	71.30
Zn	71.23	69.90	67.09

**Table A2:** Excerpt of the multiple regressions variance analyses.

Variable	Contribution in %	*p* Value
pH regression	81.76	0.000
pH_rain_	41.95	0.000
1/t_ex_	39.80	0.000
calcium regression	85.76	0.000
runoff	85.76	0.000
zinc regression	71.23	0.000
t_ex_^−2^	0.85	0.000
T/t_con_	14.43	0.002
RH ∗ t_con_	43.86	0.000
Zn_b_ ∗ runoff ∗ t_con_	4.23	0.000
runoff^2^ ∗ t_con_	4.25	0.000
RH ∗ t_con_^2^	0.13	0.011
(Zn_b_ ∗ runoff ∗ t_con_)/tex	3.48	0.000
vanadium regression	80.80	0.000
T	2.12	0.000
t_ex_ ∗ T	37.73	0.000
T/t_ex_	30.02	0.000
t_ex_^2^/t_con_	1.30	0.000
RH/t_ex_^2^	0.93	0.000
1/t_ex_^2^t_con_	6.44	0.000
1/t_ex_ t_con_^2^	2.27	0.000
